# Vasoproliferative Retinal Tumor with Hemangioblastoma-like Features: Evaluation with von Wilebrand Factor

**DOI:** 10.3390/jcm15124440

**Published:** 2026-06-08

**Authors:** Daiki Kuraoka, Hiromasa Hirai, Yu Morimoto, Kazuya Sakai, Akihiko Yoshizawa, Satoru Kase

**Affiliations:** 1 Department of Ophthalmology, Nara Medical University, Kashihara 8348521, Japan; k159378@naramed-u.ac.jp (D.K.); hirai-masa@naramed-u.ac.jp (H.H.); k177476@naramed-u.ac.jp (Y.M.); 2 Department of Blood Transfusion Medicine, Nara Medical University, Kashihara 8348521, Japan; ks13122@naramed-u.ac.jp; 3 Department of Diagnostic Pathology, Nara Medical University, Kashihara 8348521, Japan; akyoshi@naramed-u.ac.jp

**Keywords:** vasoproliferative retinal tumor, retinal hemangioblastoma, von Willebrand factor, vitreous fluid, immunohistochemistry

## Abstract

**Objectives**: To investigate the clinicopathologic characteristics and molecular biomarkers of atypical vasoproliferative retinal tumor (VPRT) with hemangioblastoma-like histopathologic features and concomitant von Willebrand factor (VWF) abnormalities. **Methods**: A 48-year-old woman undergoing phacoemulsification and 25-gauge pars plana vitrectomy with tumor resection was evaluated. Histopathological findings and immunohistochemical study of the resected tumor were performed using CD34, α-smooth muscle actin (αSMA), and glial fibrillary acidic protein (GFAP) markers. Preoperative plasma and intraoperative vitreous fluid VWF antigen levels, as well as ristocetin cofactor activity, were quantified using latex immunoturbidimetry. **Results**: Ultra-widefield imaging and angiography demonstrated a peripheral retinal tumor with intense vascular leakage and surrounding capillary nonperfusion. Histopathology showed hyalinized vascular components supportive of VPRT, along with abundant CD34/α-SMA-positive microvessels and scant GFAP-positive glial cells. Notably, numerous foamy vacuolated poorly differentiated cells suggested mixed hemangioblastoma-like features. Preoperative plasma VWF antigen (182.6%) and ristocetin cofactor activity (147.7%) were elevated, and vitreous VWF antigen was successfully detected at a low but distinct level (7.7%).and suggests that VWF abnormalities in the plasma and vitreous may reflect endothelial activation and/or blood–retinal barrier disruption in a subset of vascularized retinal tumors. **Conclusions**: Our findings demonstrate that VPRT may exhibit mixed clinicopathologic features, including hemangioblastoma-like components, which underscores the necessity of immunohistochemical assessment for definitive diagnosis. Furthermore, the quantification of VWF abnormalities in the plasma and vitreous suggests that VWF serves as a potential biomarker reflecting endothelial activation and/or blood–retinal barrier disruption in vascularized retinal tumors.

## 1. Introduction

Vasoproliferative retinal tumor (VPRT) is a benign peripheral retinal tumor that may be complicated by exudation, vitreous opacity/hemorrhage, epiretinal membrane (ERM) formation, and secondary macular changes leading to visual impairment [1,2]. While traditionally considered a vasoproliferative tumor, VPRT has also been proposed to represent a reactive process with prominent astrocytic/glial proliferation (reactive retinal astrocytic tumor; RRAT or focal nodular gliosis) accompanied by hyalinized vessels and ghost vessel-like vascular structures. These pathological features suggest that VPRT may encompass a spectrum of lesions with variable proportions of vascular and glial elements [3,4]. Retinal hemangioblastoma is another common retinal vascular tumor, typically associated with von Hippel-Lindau (VHL) disease. The histopathological findings are characterized by capillary proliferation and stromal tumor cells, the latter of which may have vacuolated/foamy cytoplasm [5,6].

von Willebrand factor (VWF) is a multimeric glycoprotein released mainly from vascular endothelial cells and megakaryocytes and is widely recognized as a biomarker of endothelial activation and injury [7]. Elevated plasma VWF levels have been reported in several thrombotic and inflammatory vascular disorders and may reflect vascular endothelial dysfunction and microvascular stress [8,9,10]. Emerging evidence, including studies from our group, suggests that the VWF- a thrombospondin type 1 motif, member 13 (ADAMTS13) axis may be involved in the pathophysiology of ocular vascular diseases. In pachychoroid neovasculopathy, unusually large VWF multimers were observed more frequently than in age-related macular degeneration (AMD), supporting the concept that impaired VWF processing contributes to ocular microvascular dysfunction and hemorrhagic manifestations [11]. Furthermore, we previously demonstrated that, in branch retinal vein occlusion (BRVO) with cystoid macular edema, plasma VWF levels were associated with retino-choroidal structural parameters and with decrease in the levels significantly after anti-vascular endothelial growth factor (VEGF) therapy, indicating that VWF may serve as a marker of endothelial activation and vascular injury in retinal vascular diseases [12]. However, to our knowledge, there have been no previous reports evaluating VWF levels in intraocular tumors.

Therefore, the purpose of this study was to evaluate the clinicopathologic characteristics and molecular biomarkers of an atypical VPRT. Here, we investigate a case of VPRT subjected to surgical resection, demonstrating mixed histopathologic features compatible with VPRT alongside concomitant hemangioblastoma-like findings. Furthermore, to elucidate the underlying pathophysiology, VWF levels were quantified in the plasma and in the vitreous fluid obtained intraoperatively, and compared against control eyes.

## 2. Materials and Methods

### 2.1. Subject and Clinical Evaluation

The clinical data of a female patient with an atypical retinal vascular tumor who underwent surgical intervention at our institution were retrospectively reviewed. Comprehensive ophthalmic examinations were performed, including best-corrected visual acuity (BCVA) using a standard Snellen chart, intraocular pressure (IOP) measurement, slit-lamp biomicroscopy, and dilated fundus examination. Multimodal imaging was conducted, including ultra-widefield fundus photography, fluorescein angiography (FA), and indocyanine green angiography (ICGA) using a scanning laser ophthalmoscope (Optos, Dunfermline, UK). Optical coherence tomography (OCT) (Spectralis; Heidelberg Engineering, Heidelberg, Germany) was utilized to evaluate macular pathology and subretinal fluid.

### 2.2. Surgical Procedure and Vitreous Sampling

To address the peripheral vascular lesion and associated macular complications under general anesthesia due to comorbid schizophrenia, a combination of phacoemulsification, intraocular lens implantation, and 25-gauge pars plana vitrectomy (PPV) was performed. Prior to any intraocular infusion, an undiluted vitreous sample was collected at the initiation of the vitrectomy. Core vitrectomy, epiretinal membrane (ERM) peeling, and surgically induced posterior vitreous detachment were performed. The peripheral reddish-orange lesion was resected using 25-gauge scissors and forceps, and extracted through the scleral incision after making an opening in the posterior capsule. Intraoperative arterial hemorrhage encountered during tumor manipulation was controlled using a vitreous cutter. Endolaser photocoagulation was applied around the retinectomy site, followed by silicone oil tamponade. Subsequent surgeries, including silicone oil removal, repeat PPV, encircling scleral buckling, and sulfur hexafluoride (SF6) gas tamponade, were documented during the clinical course.

### 2.3. Histopathologic and Immunohistochemical Analyses

The resected tumor tissue was fixed in 10% neutral-buffered formalin, embedded in paraffin, and cut into serial sections. Sections were stained with hematoxylin and eosin (H&E) for morphologic assessment. Immunohistochemical staining was performed using an automated immunostaining system according to the manufacturer’s standard protocols. The primary antibodies used were as follows: anti-CD34 (clone QBEnd/10; Agilent Technologies, Santa Clara, CA, USA), anti-α-smooth muscle actin (αSMA; clone 1A4; Agilent Technologies), and anti-glial fibrillary acidic protein (GFAP; clone 6F2; Agilent Technologies). The immunostaining procedure, including 3,3′-diaminobenzidine (DAB) chromogen development and hematoxylin counterstaining, was fully automated. Stained sections were examined and imaged using a BZ-9000 microscope (Keyence, Osaka, Japan).

### 2.4. Measurement of von Willebrand Factor

Plasma and vitreous von Willebrand Factor (VWF) assays preoperative plasma and intraoperative undiluted vitreous samples were harvested. Plasma and vitreous VWF antigen (VWF:Ag) and VWF ristocetin cofactor activity (VWF:RCo) were measured on a CN-3000 automated coagulation analyzer (Sysmex, Kobe, Japan) using Siemens Healthineers (Siemens, Erlangen, Germany) reagents. VWF:Ag was determined by a latex immunoturbidimetric assay, and VWF:RCo by a ristocetin-induced platelet agglutination assay using fixed platelets. The analytical measuring ranges for these assays were 2–600% and 10–400%, respectively. Vitreous VWF:Ag quantification was achieved using the latex immunoturbidimetry (latex agglutination-based method). For comparison, undiluted vitreous samples obtained from two patients with idiopathic ERM without retinal vascular tumors were analyzed as negative controls.

## 3. Results

### 3.1. Baseline Clinical Presentation and Multimodal Imaging

**Figure 1:**
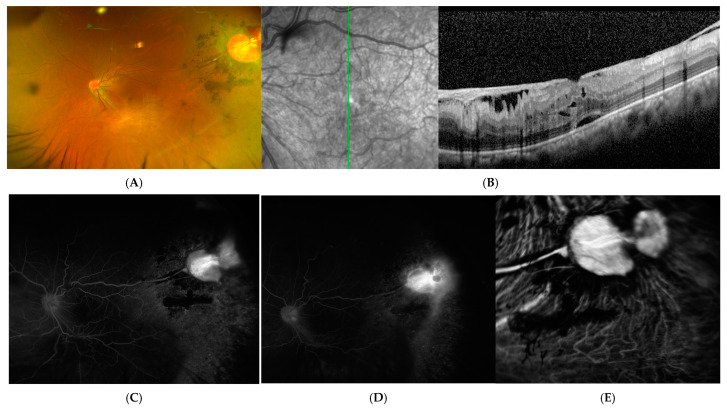
Preoperative multimodal imaging of the left eye. (**A**) Ultra-widefield fundus photograph showing an elevated reddish-orange retinal lesion measuring approximately four-disc diameters in size, with a smaller secondary reddish lesion (approximately two-disc diameters) located adjacent to the main tumor further in the periphery. (**B**) OCT image displaying mild subretinal fluid accompanied by a thickened posterior hyaloid membrane. The left panel is an infrared light, showing vertical section of the macula as a green line indicated. (**C**) Early-phase FA identifying a prominent feeding vessel extending from the peripapillary region of the optic disc. (**D**) Late-phase FA demonstrating faint peritumoral fluorescein leakage with surrounding capillary nonperfusion; note that part of the peritumoral hyperfluorescence is consistent with a window defect secondary to retinal pigment epithelium atrophy. (**E**) ICGA revealing intense hyperfluorescence of the tumor proper contrasted with peritumoral hypofluorescence.

A 48-year-old woman with a history of schizophrenia and prior retinal laser treatment presented with decreased vision in her left eye. The baseline BCVA was 20/16 OD and 20/70 OS. IOP was 12 mmHg in both eyes. Fundus examination of the left eye revealed vitreous opacity, an ERM, and an elevated reddish-orange peripheral retinal lesion measuring approximately four-disc diameters in the superotemporal fundus, adjacent to a smaller secondary reddish lesion (Figure 1A). OCT imaging displayed mild subretinal fluid with a thickened posterior hyaloid membrane (Figure 1B). Early-phase FA identified a prominent feeding vessel extending from the peripapillary region of the optic disc (Figure 1C: arrow). Late-phase FA demonstrated faint peritumoral fluorescein leakage, surrounding capillary non-perfusion, and a window defect secondary to retinal pigment epithelium atrophy (Figure 1D). ICGA confirmed hyperfluorescence of the tumor proper with surrounding peritumoral hypofluorescence (Figure 1E).

### 3.2. Surgical Outcomes and Postoperative Course

**Figure 2:**
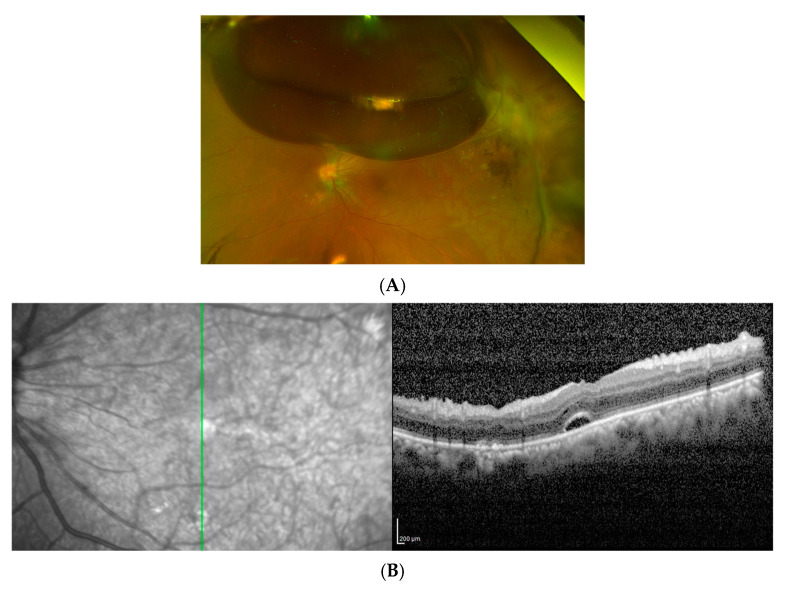
Postoperative multimodal imaging after the secondary surgery. (**A**) Ultra-widefield fundus photograph showing complete removal of the tumor, with no apparent hemorrhage or retinal detachment, and the presence of residual intraocular SF6 gas. (**B**) Postoperative OCT image demonstrating that mild subretinal fluid remains, but the thickened posterior hyaloid membrane has been completely removed and the macular traction has been fully released. The left panel is an infrared light, showing vertical section of the macula as a green line indicated.

Following the primary combined surgery, retinal re-attachment was initially maintained; however, a transient elevation of IOP occurred in Month X + 3, which was stabilized with topical and oral antiglaucoma medications. In Month X + 4, due to a secondary retinal detachment, the subject underwent silicone oil removal, repeat PPV, encircling scleral buckling, and SF6 gas tamponade (Figure 2A,B). At 2 weeks following the final intervention, the BCVA stabilized at 20/70 OS.

### 3.3. Histopathologic Findings

**Figure 3:**
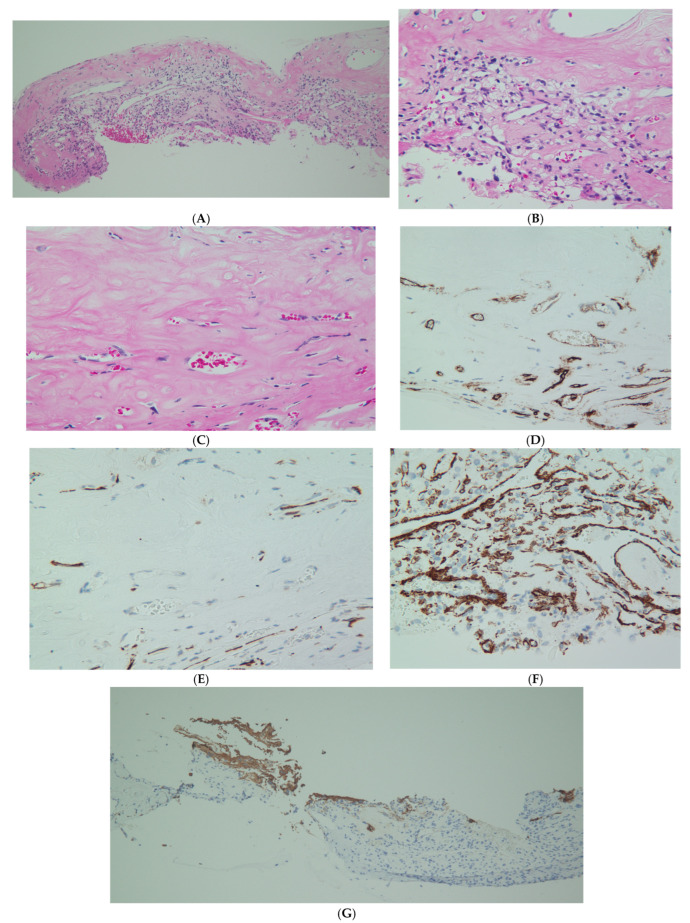
Histopathologic and immunohistochemical analyses of the resected peripheral retinal tumor. (**A**) Low-power and (**B**,**C**) high-power H&E staining demonstrating that the retinal tumor adjacent to the main lesion consists of abundant cellular components, fine vascular lumina, and amorphous hyalinized areas. (**D**) Immunohistochemical staining for CD34 revealing numerous positive microvessels throughout the lesion. (**E**,**F**) Immunohistochemical staining for αSMA in the microvessels, demonstrating heterogenous immunoreactivity with distinctly negative and positive areas. (**G**) Immunohistochemical staining for GFAP showing only a few scattered positive glial cells.

H&E staining of the resected tissue revealed abundant cellular components, fine vascular lumina, and amorphous hyalinized areas (Figure 3A–C). Immunohistochemistry demonstrated that numerous microvessels were strongly positive for CD34 (Figure 3D). In contrast, αSMA staining showed a mixed pattern of positive and negative immunoreactivity (Figure 3E,F). Notably, numerous poorly differentiated cells with foamy (vacuolated) cytoplasm were intermingled within the lesion, corresponding to the αSMA-positive regions. GFAP-positive glial cells were scant throughout the resected tumor sections. These findings collectively supported a diagnosis consistent with VPRT on a background of hyalinized vascular elements, accompanied by mixed hemangioblastoma-like features.

### 3.4. VWF Biomarker Quantifications

Quantitative analysis of the molecular biomarkers yielded distinct abnormalities. Preoperative plasma testing revealed elevated levels of VWF:Ag at 182.6% and VWF:RCo at 147.7%. In the surgical vitreous specimen, VWF:Ag was quantifiably detectable at 7.7%. In stark contrast, VWF:Ag levels were completely undetectable in the vitreous samples obtained from the two control patients with idiopathic ERM.

## 4. Discussion

This study investigated a retinal vascular lesion presenting with vitreous opacity and macular pathology (subretinal fluid and ERM) together with a peripheral reddish elevated subretinal lesion. In such presentations, the differential diagnosis includes VPRT, retinal hemangioblastoma (including VHL-associated lesions), choroidal hemangioma, metastatic tumors, and inflammatory granulomatous lesions [1,2,5]. VPRT typically appears as a yellow-red to red peripheral elevated lesion and may cause visual impairment primarily through exudation and tractional sequelae, including ERM and secondary macular changes [1,2]. In a large clinical series, VPRT comprised both primary and secondary forms, with secondary tumors more frequently associated with other ocular pathologies and potentially differing in clinical behavior [3]. Management options for VPRT depend on lesion size, exudation severity, macular involvement, and the presence of vitreous opacity or traction, and include observation, laser photocoagulation, cryotherapy, radiotherapy, intravitreal anti-VEGF therapy, and vitreoretinal surgery [1,2,13]. When vitreous opacity limits adequate assessment and local therapies, or when macular complications significantly contribute to visual loss, vitreoretinal surgery can be appropriate [1,13]. In the targeted lesion, tumor resection served both therapeutic and diagnostic purposes by removing a presumed source of exudation/traction and providing tissue for definitive pathologic assessment. In addition, because the patient had schizophrenia, treatment under local anesthesia was considered difficult. As repeated general anesthesia was undesirable, tumor resection was initially performed during the same surgery.

The major diagnostic challenge in this pathology was the distinction between VPRT and retinal hemangioblastoma. Clinically, the presence of a feeder vessel and angiographic leakage raised the possibility of retinal hemangioblastoma. However, the peripheral location, vitreous opacity, associated epiretinal membrane, and exudative/tractional macular changes were also characteristic of VPRT. On FA, early-phase imaging demonstrated a feeder vessel extending from the peripapillary region, whereas late-phase imaging showed faint peritumoral leakage and surrounding capillary nonperfusion. In addition, part of the peritumoral hyperfluorescence on fluorescein angiography was considered consistent with a window defect due to retinal pigment epithelium atrophy. On ICGA, the tumor itself showed hyperfluorescence, whereas the peritumoral area showed hypofluorescence. Although these angiographic findings were informative, they were not sufficiently specific to establish a definitive diagnosis, and therefore histopathologic confirmation remained essential.

Optical coherence tomography angiography (OCTA) may also provide useful complementary information in the evaluation of retinal vascular tumors. In VPRT, widefield OCTA has been reported to detect intratumoral flow signals and may be useful not only for diagnosis but also for monitoring treatment response [14]. In retinal hemangioblastoma, particularly in lesions located near the optic disc or along the vascular arcades, OCTA has also been reported to be useful for lesion detection and visualization of vascular morphology [15]. Although OCTA was not performed in the present case, it might have provided additional noninvasive information regarding the vascular architecture of the lesion and therefore could have been worth attempting. In particular, if image acquisition had been feasible, OCTA might have served as an adjunctive tool in the differential diagnosis and in the longitudinal assessment of treatment-related vascular changes.

The central feature of this investigation is its histopathology. While VPRT has been described clinically as a vasoproliferative tumor, some lesions categorized as VPRT may represent a spectrum of reactive processes with variable proportions of glial and vascular elements [1,4,16]. In the current resected tissue, vascular structures exhibited areas of marked hyalinization of vessel walls as well as cellular proliferation with fine vascular lumina, the vascular morphology of which typically associated with VPRT. Moreover, immunohistochemical staining showed CD34-positive (endothelial marker) and αSMA-negative (pericyte/smooth muscle marker), a pattern regarded as characteristic of, and supportive of, VPRT-associated vascular changes [1,4]. Furthermore, numerous poorly differentiated cells with foamy (vacuolated) cytoplasm were intermingled within the lesion. These findings resembled histopathologic features of tumor cells described in retinal hemangioblastoma associated with VHL disease [5,6]. In addition, the lesion contained abundant microvessels positive for both CD34 and αSMA and areas with relatively scant GFAP-positive glial cells, supporting a hemangioblastoma-like component within a lesion otherwise consistent with VPRT. Previous immunohistochemical studies of hemangioblastoma have shown that GFAP-positive cells may be present within the lesion; however, such GFAP immunoreactivity is generally interpreted as representing entrapped or reactive glial cells rather than stromal tumor cells themselves. Jurco et al. reported that stromal cells in hemangioblastoma were consistently negative for GFAP, supporting the view that GFAP is not a specific marker of stromal tumor cell differentiation. Therefore, the relatively scant GFAP-positive glial cells in the present case do not exclude hemangioblastoma-like differentiation but rather indicate that the glial component was limited [7]. Taken together, the present findings provide the first histopathologic documentation of a retinal vascular tumor exhibiting features of both entities. Although there was no systemic history suggestive of VHL disease, retinal hemangioblastoma remains an important differential diagnosis for vascular-appearing peripheral retinal lesions.

VWF is a large multimeric glycoprotein synthesized and secreted mainly by vascular endothelial cells and plays an essential role in primary hemostasis by mediating platelet adhesion to injured vessels and stabilizing coagulation factor VIII [16,17]. VWF is specifically cleaved by a disintegrin and metalloproteinase with ADAMTS13, and the balance between VWF and ADAMTS13 is important for maintaining systemic hemostatic function [17]. Because the release of VWF increases in response to endothelial activation and injury, elevated plasma VWF levels are widely regarded as a biomarker of endothelial dysfunction and have been reported in hypertension, atherosclerosis, and several thrombotic vascular disorders [8,9,10,18,19]. In the ophthalmic field, increased plasma VWF antigen levels have also been described in age-related macular degeneration, and unusually large VWF multimers have been detected more frequently in pachychoroid neovasculopathy, supporting the relevance of the VWF–ADAMTS13 axis to ocular vascular dysfunction [10,11,12]. We have previously shown that impaired VWF processing contributes to ocular microvascular dysfunction and hemorrhagic manifestations in AMD and BRVO [11,12]. In the present analysis, preoperative plasma VWF antigen and ristocetin cofactor activity were elevated, and vitreous VWF antigen was detectable at a low level, whereas these findings were not observed in control eyes with ERM without VPRT. These findings may be consistent with local endothelial stress in a vascularized retinal lesion, particularly given the marked angiographic leakage, surrounding capillary nonperfusion, and the presence of CD34-positive/αSMA-negative vessels suggesting reduced mural support of the vascular wall. Although evaluating a single affected subject is insufficient to establish a disease-specific mechanistic role for VWF in either VPRT or retinal hemangioblastoma, the quantified VWF abnormality should be regarded as an exploratory biomarker finding that may reflect endothelial activation and/or blood–retinal barrier disruption. To advance future research directions, broader quantitative investigations and a larger cohort analysis will be required to determine whether VWF activation is a reproducible feature of such lesions and whether it can serve as a diagnostic discriminator.

Comparative pathology may also provide a useful perspective on the vascular biology of this lesion. In a recent case series of canine hemangioblastoma, endothelial cells were consistently immunolabeled with VWF, whereas stromal cells were negative for VWF, supporting the concept that hemangioblastoma contains a prominent endothelial vascular component rather than diffuse stromal VWF expression [20]. In the present case, the elevated plasma VWF levels and detectable vitreous VWF may therefore be interpreted cautiously as exploratory evidence of endothelial activation and/or endothelial injury in a retinal vascular tumor. Although this single case does not allow any disease-specific conclusion, it raises the possibility that VPRT with hemangioblastoma-like features and hemangioblastoma may share, at least in part, a common endothelial stress response or blood–retinal barrier dysfunction. This interpretation remains hypothesis-generating and warrants further investigation in additional cases.

Overall, this study highlights the diagnostic value of tissue assessment in peripheral retinal tumor-like lesions requiring surgery and suggests that lesions clinically consistent with VPRT may show mixed histopathologic features that have implications for classification and pathophysiologic understanding [1,4,21].

## 5. Conclusions

Our findings demonstrate that peripheral reddish elevated retinal tumors can present with histopathologic findings compatible with VPRT alongside hemangioblastoma-like features, which underscores the importance of detailed histopathologic assessment when clinical differentiation is challenging. Furthermore, the detected VWF abnormality may reflect endothelial dysfunction and contribute to the pathophysiology of VPRT.

## Data Availability

All relevant data supporting the findings of this study are included in the article. Additional data are available from the corresponding author upon reasonable request. Further data are not publicly available due to privacy restrictions.

## Abbreviations

The following abbreviations are used in this manuscript:

αSMA	alpha-smooth muscle actin
ADAMTS13	a thrombospondin type 1 motif, member 13
AMD	age-related macular degeneration
BCVA	best-corrected visual acuity
BRVO	branch retinal vein occlusion
ERM	epiretinal membrane
FA	fluorescein angiography
GFAP	glial fibrillary acidic protein
H&E	hematoxylin and eosin
ICGA	indocyanine green angiography
IOL	intraocular lens
NPA	nonperfusion area
OCT	optical coherence tomography
OD	oculus dexter
OS	oculus sinister
PVD	posterior vitreous detachment
PPV	pars plana vitrectomy
RRAT	reactive retinal astrocytic tumor
SF6	sulfur hexafluoride
VEGF	vascular endothelial growth factor
VHL	von Hippel-Lindau
VPRT	vasoproliferative retinal tumor
VWF	von Willebrand factor
VWF:Ag	von Willebrand factor antigen
VWF:RCo	von Willebrand factor ristocetin cofactor activity

## References

[1] Heimann H., Bornfeld N., Vij O., Coupland S.E., Bechrakis N.E., Kellner U., Foerster M.H. (2000). Vasoproliferative tumours of the retina. Br. J. Ophthalmol..

[2] Damato B. (2006). Vasoproliferative retinal tumour. Br. J. Ophthalmol..

[3] Shields C.L., Kaliki S., Al-Dahmash S., Rojanaporn D., Shukla S.Y., Reilly B., Shields J.A. (2013). Retinal vasoproliferative tumors: Coparative clinical features of primary vs secondary tumors in 334 cases. JAMA Ophthalmol..

[4] Perry L.J.P., Jakobiec F.A., Zakka F.R., Reichel E., Herwig M.C., Perry A., Brat D.J., Grossniklaus H.E. (2013). Reactive retinal astrocytic tumors (so-called vasoproliferative tumors): Histopathologic, immunohistochemical, and genetic studies of four cases. Am. J. Ophthalmol..

[5] Dollfus H., Massin P., Taupin P., Nemeth C., Amara S., Giraud S., Béroud C., Dureau P., Gaudric A., Landais P. (2002). Retinal hemangioblastoma in von Hippel-Lindau disease: A clinical and molecular study. Investig. Ophthalmol. Vis. Sci..

[6] Miyazawa A., Inoue M., Hirakata A., Okada A.A., Iihara K., Fujioka Y. (2009). Expression of inhibin α by stromal cells of retinal angiomas excised from a patient with von Hippel-Lindau disease. Jpn. J. Ophthalmol..

[7] Jurco S., Nadji M., Harvey D.G., Parker J.C., Font R.L., Morales A.R. (1982). Hemangioblastomas: Histogenesis of the stromal cell studied by immunocytochemistry. Hum. Pathol..

[8] Blann A.D., Naqvi T., Waite M., McCollum C.N. (1993). von Willebrand factor and endothelial damage in essential hypertension. J. Hum. Hypertens..

[9] Blann A.D., McCollum C.N. (1994). von Willebrand factor, endothelial cell damage and atherosclerosis. Eur. J. Vasc. Surg..

[10] Lip G.Y., Blann A. (1997). von Willebrand factor: A marker of endothelial dysfunction in vascular disease. Cardiovasc. Res..

[11] Hirai H., Yamashita M., Matsumoto M., Hayakawa M., Sakai K., Ueda T., Ogata N. (2021). Analysis focusing on plasma von Willebrand factor in pachychoroid neovasculopathy and age-related macular degeneration. Sci. Rep..

[12] Hirai H., Yamashita M., Matsumoto M., Nishiyama T., Wada D., Okabe N., Mizusawa Y., Jimura H., Ueda T., Ogata N. (2022). Alteration of plasma von Willebrand factor in the treatment of retinal vein occlusion with cystoid macular edema. PLoS ONE.

[13] Pereira A.F., Teixeira-Martins R., Rocha-Sousa A., Penas S. (2025). Vasoproliferative retinal tumors: Manifestations, management, and Outcomes in a Case Series. Case Rep. Ophthalmol..

[14] Tanimukai T., Noda K., Hirooka K., Kase S., Ishida S. (2022). Noninvasive Imaging of a Vasoproliferative Retinal Tumor Treated with Cryopexy. Case Rep. Ophthalmol..

[15] Takahashi A., Muraoka Y., Koyasu S., Arakawa Y., Nakamura E., Tsujikawa A. (2023). Novel Manifestation of Retinal Hemangioblastomas Detected by OCT Angiography in von Hippel-Lindau Disease. Ophthalmology.

[16] Sedler J.E. (1998). Biochemistry and genetics of von Willebrand factor. Annu. Rev. Biochem..

[17] Crawley J.T.B., de Groot R., Xiang Y., Luken B.M., Lane D.A. (2011). Unraveling the scissile bond: How ADAMTS13 recognizes and cleaves von Willebrand factor. Blood.

[18] Buchtele N., Schwameis M., Gilbert J.C., Schörgenhofer C., Jilma B. (2018). Targeting von Willebrand Factor in ischaemic stroke: Focus on clinical evidence. Thromb. Haemost..

[19] Edvardsen M.S., Hindberg K., Hansen E.-S., Morelli V.M., Ueland T., Aukrust P., Brækkan S.K., Evensen L.H., Hansen J.-B. (2021). Plasma levels of von Willebrand factor and future risk of incident venous thromboembolism. Blood Adv..

[20] Aytaş Ç., Cauduro A., Falzone C., Gianni S., Tomba A., Cantile C. (2025). Canine Hemangioblastoma: Case Series and Literature Review. Animals.

[21] Hashimoto I., Takase H., Kase S., Iwasaki Y., Kobayashi D., Ohno-Matsui K. (2024). Clinicopathological analysis of secondary retinal vasoproliferative tumor/reactive retinal astrocytic tumor successfully treated by endoresection. Retin. Cases Brief Rep..

